# Use of Artificial Intelligence in Burn Assessment: A Scoping Review with a Large Language Model-Generated Decision Tree

**DOI:** 10.3390/ebj7010004

**Published:** 2026-01-04

**Authors:** Sebastian Holm, Fredrik Huss, Bahaman Nayyer, Johann Zdolsek

**Affiliations:** 1 Department of Plastic and Reconstructive Surgery, Örebro University Hospital, Faculty of Medicine and Health, Örebro University, 70182 Örebro, Sweden; 2 Department of Surgical Sciences, Plastic Surgery, Uppsala University, 75185 Uppsala, Sweden; 3 Burn Center, Department of Plastic and Maxillofacial Surgery, Uppsala University Hospital, 75185 Uppsala, Sweden; 4 School of Medical Sciences, Faculty of Medicine and Health, Örebro University, 70182 Örebro, Sweden

**Keywords:** artificial intelligence, burn assessment, deep learning, convolutional neural networks, large language models, decision support, segmentation, burn depth, burn area, treatment recommendation

## Abstract

**Background:** Burns cause about 180,000 deaths annually and lead to substantial morbidity, especially in low- and middle-income countries. Clinical assessment of burn depth and TBSA relies on visual and bedside examination and remains subjective. Convolutional neural networks (CNNs) have been proposed to improve objectivity in image-based burn assessment, but clinical generalizability and acceptance remain uncertain. **Aims:** To map current evidence on CNN performance for burn TBSA, burn depth and treatment-related tasks and to explore whether a large language model (LLM) can organize extracted findings into a transparent, literature-derived orientation decision tree. **Methods:** We performed a scoping review following PRISMA-ScR. PubMed, Web of Science and Cochrane were searched on 5 April 2025. Eligible studies reported CNN analysis of 2D burn images and quantitative performance metrics. We summarized reported values descriptively. We then provided a structured summary of extracted findings to ChatGPT to draft a one-page orientation decision tree. Two consultant burn surgeons reviewed the figure for clarity and plausibility. **Results:** Of 659 records, 24 studies were included. Across studies, reported performance for TBSA and depth assessment was often high, but study designs, datasets, labels, imaging modalities and validation strategies varied substantially. High reported performance does not necessarily imply clinical robustness or real-world accuracy. A single study reported high test-set accuracy for graft versus non-graft using heavily expanded data. This value should not be generalized. **Conclusions:** CNNs show promise for image-based burn TBSA and depth assessment, but heterogeneity, dataset limitations and limited external validation restrict interpretation and clinical transfer. The LLM-derived decision tree is a literature-synthesis orientation figure, not a clinical decision-support tool.

## 1. Introduction

Burns are a global health problem, causing about 180,000 deaths annually [1]. Most burn injuries occur in low- and middle-income countries (LMICs) [2]. Global overviews indicate that nearly two-thirds of burn injuries arise in the African and South-East Asia regions, and burns are among the leading causes of disability-adjusted life years (DALYs) lost in LMICs [1].

Clinical burn depth assessment relies on inspection and examination. Clinicians assess colour, capillary refill, pinprick or sensation and injury history or mechanism. This remains subjective. Compared with histopathology, specialist burn depth assessment is correct in about 70 to 80% of cases [3]. Accuracy is lower among less experienced clinicians [3]. In contrast, laypeople aided by assessment tools have been reported to estimate TBSA more accurately and consistently than burn professionals using the Rule of Palm and the Lund–Browder chart [4].

Recent advancements in artificial intelligence have led to systems designed to support burn care. Deep learning includes artificial neural networks used for image analysis, including convolutional neural networks (CNNs) [5]. In burn image analysis, CNNs are widely used. They learn patterns through layered processing of image data. They can identify features such as texture, shape and colour across large image sets. Different CNNs share common building blocks, but architectures differ in how layers are arranged and connected (e.g., depth, kernel sizes, skip connections, encoder–decoder designs).

Several CNN architectures have achieved strong performance in medical imaging, including AlexNet, VGG, U-Net, ResNet and DenseNet, as described in recent reviews of deep learning in medical imaging [6]. Performance depends heavily on data quality and training approach. Attention modules, such as Bidirectional Associative Memory (BAM), can be integrated into these backbones [7,8]. These architectures can be trained by datasets from scratch or by the use of pretrained models that use transfer learning, which is then further trained with a smaller training set [5]. One method to increase the medical images in the training set is by using Generative Adversarial Networks (GANs) that attempt to produce photorealistic images of burn wounds [9].

To evaluate CNN performance for TBSA and burn depth tasks, studies report different metrics. These include *diagnostic accuracy*, *recall*, *precision*, *Dice coefficient* (DC), *specificity*, *Intersection over Union* (IoU) and many more. *Diagnostic accuracy* refers to the ability of a CNN to correctly classify burn depth or delineate injured skin for TBSA estimation [10]. *Recall* and *precision* analyze the same thing but from different perspectives; *recall* reflects how many true positives are identified. *Precision* reflects how many predicted positives are true positives. Although *recall* and *precision* are quite similar, they are both important for the information they give. To optimize recall and precision, they are combined in the Dice coefficient, defined as 2 x precision x recall divided by precision plus recall (2PR/(P + R)). *Specificity* reflects the number of predicted negative cases that are true negatives. It thus describes the test’s ability to recognize healthy skin [10]. The *Intersection over Union* analyzes the test’s segmentation capabilities. Here, it is a measure of how well the CNN determines %TBSA. It is similar to the *Dice coefficient* in that both quantify overlap between predicted and ground-truth segmentation masks. In fact, for binary segmentation the *Dice coefficient* = 2 × IoU/(1 + IoU) [11].

The aim of this study was to evaluate the current evidence for artificial intelligence, specifically CNNs, in the assessment of burn TBSA, burn depth and treatment-related prediction tasks (e.g., surgery vs. non-surgery, graft vs. non-graft and healing-time category prediction) rather than treatment efficiency. In addition, to evaluate one large language model’s (LLM) (ChatGPT, Version 5.0) ability to generate clinical decision trees based on this scoping review.

## 2. Methods

This scoping review followed the PRISMA extension for scoping reviews (PRISMA-ScR) checklist to ensure transparent reporting and methodological rigor [12]. No protocol was pre-registered as PROSPERO does not currently accept scoping reviews [13]. In accordance with PRISMA-ScR guidance, we did not perform a formal risk-of-bias assessment using a single appraisal tool due to heterogeneity in designs, datasets and outcome metrics.

The following inclusion and exclusion criteria were used to gather studies.

Inclusion criteria:

- Written in English.
- Reported use of CNN for analysis of two-dimensional burn images.
- Reported quantitative model performance metrics.
- Exclusion criteria:
- Not focused on burn assessment tasks (TBSA, burn depth or treatment-related tasks).
- Did not use CNN-based methods.
- Did not report quantitative performance metrics.
- Conference abstracts, editorial, letters, protocols or non-peer-reviewed records.

A comprehensive search of three databases: PubMed, Cochrane Library (Wiley) and Web of Science (Clarivate) was conducted on 5 April 2025. Search strategies were designed for each database using Boolean operators and keywords related to “burns”, “artificial intelligence” and “convolutional neural networks”. Full search strategies are provided in Supplement S1. All retrieved records were imported into Covidence systematic review software (Veritas Health Innovation, Melbourne, Australia). Duplicates were removed automatically. Blinded title and abstract screening were performed in duplicate using Covidence by two reviewers. Disagreements were resolved through discussion with a third reviewer. The same procedure was used for the full text screening.

Data extraction was performed using a standardized template. Extracted information included author, paper, year, country, study design, artificial intelligence (AI) model, dataset, training set, outcome and statistical performance metrics. All quantitative summaries in this scoping review are descriptive aggregations of reported study-level values. They are not pooled estimates and should not be interpreted as meta-analytic performance.

### LLM-Derived Decision Model

After data extraction, we compiled a structured, study-level summary containing task domain (TBSA/area, depth, treatment), imaging modality (RGB smartphone/clinical photography, LDPI or other), model family or architecture as reported by the authors and the published performance metrics already extracted into our tables. We provided this structured summary to ChatGPT version 5.0 (OpenAI, San Francisco, CA, USA) via the ChatGPT web application and instructed it to organize the extracted information into a one-page orientation decision tree. The prompt required the model to reproduce only the extracted models and extracted numeric values and not to generate new estimates, new statistics or new claims beyond the extracted dataset.

The LLM output was used to draft the layout and wording. Two consultant burn surgeons (J.Z., F.H.) independently reviewed the figure for clarity and plausibility and suggested wording changes. No patient data were used, and no clinical decisions were made. This figure is therefore a literature-synthesis visualization and not a trained or validated clinical tool. The full prompt and the consultant evaluation form are provided in Supplements S2 and S4. The model was queried using default system parameters in the ChatGPT web interface, without iterative prompt refinement or temperature adjustment, to minimize prompting bias.

The results were divided into three categories: burn area, burn depth and treatment, which included the AI’s capability to recommend appropriate treatment based on the assessments. We extracted performance metrics: *accuracy, precision, recall, Dice coefficient, specificity* and *Intersection over Union* (IoU) as outcome measures. For burn depth tasks, *specificity* was not used because the included studies did not report a meaningful negative class. The focus was on classifying depth within injured skin, not distinguishing burns from non-burns. When studies reported class-wise mean IoU *(mIoU)* instead of IoU, we recorded *mIoU* and summarized it separately rather than pooling it with IoU. For terminology, “Generated images” are synthetic samples created with generative models (e.g., GANs). “Expanded images” are augmented versions of real images (e.g., rotations, flips, crops, colour).

## 3. Results

**Figure 1:**
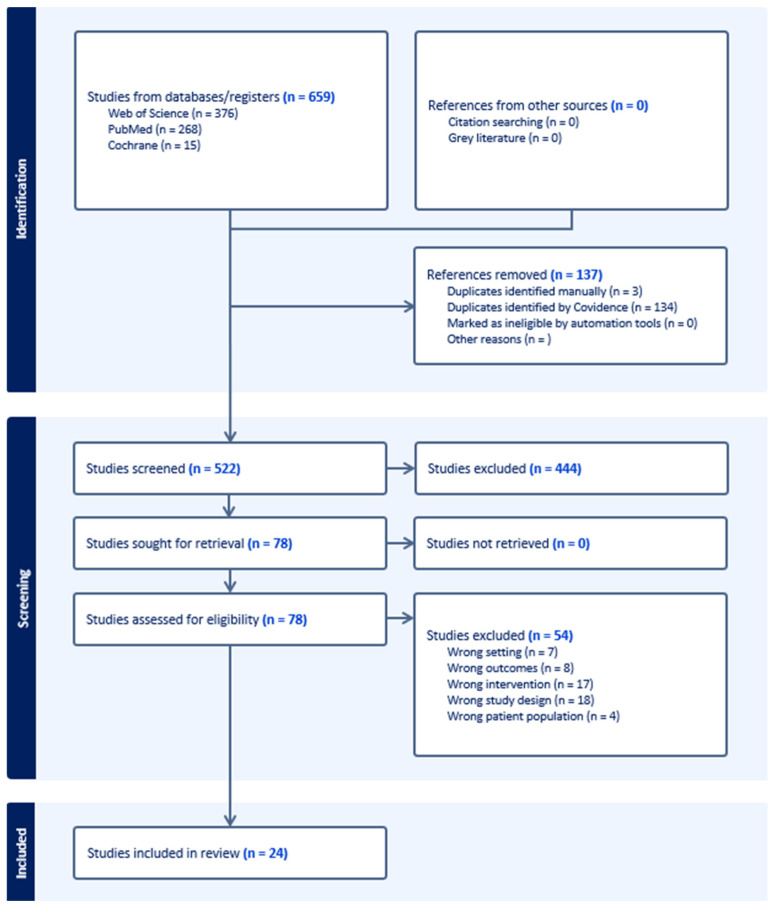
PRISMA flow chart generated from Covidence [36].

In total, the database search yielded 659 studies. After removal of 137 duplicates, 522 studies remained. The title and abstract screening were followed by full-text screening. Non-eligible studies were discarded leaving 24 studies eligible for data extraction [11,14,15,16,17,18,19,20,21,22,23,24,25,26,27,28,29,30,31,32,33,34,35]. The screening process was documented and is presented in Figure 1.

### 3.1. Summary of Included Studies

Of the 24 eligible and thus included studies, 10 included information about burn area evaluation, 17 about burn depth assessment and 4 about treatment. Most studies originated from Asia (n = 15, 62.5%), followed by Europe (n = 7, 29.1%) and North America (n = 2, 8.3%). The distribution between comparative and experimental studies was even among the included studies. All studies, but one (2019), were published after the year 2020. The number of studies in “reported outcome” is greater than the total number of studies, as some studies included information on more than one outcome. Details of each study, including results, are found under the corresponding subheading depending on which of the three categories they focused on, see Table 1.

### 3.2. Burn Area

**Figure 2:**
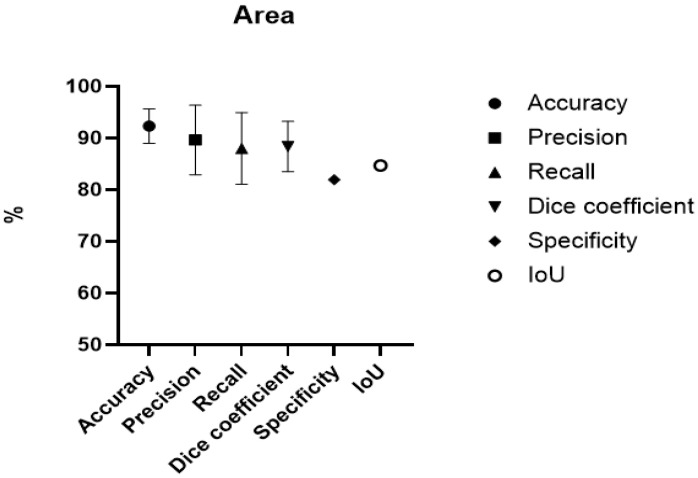
Error-bar chart showing descriptive mean values and standard deviations (SD) of reported metrics across included studies for burn area tasks. Bars represent between-study variability in reported values and do not represent uncertainty, confidence intervals or model-level precision.

In total, 11,768 images were collected and analysed using CNNs across nine different studies to help assess the burn area [11,14,15,16,17,18,19,20,21]. Of these images, 184 were Laser Doppler Imaging (LDPI) images and 1200 images were acquired through expansion. LDPI captures physiologic perfusion-related signals and is not directly comparable with RGB photography. Reported performance values across LDPI and RGB studies should therefore not be interpreted as comparative. All ten studies used %TBSA to determine the area of the burn. Different metrics were reported: *accuracy, precision, recall, Dice coefficient, specificity and* IoU. Reported descriptive means and ranges in this review span multiple imaging modalities and study designs, and they are presented for overview purposes only. Across burn-area studies, the descriptive mean *accuracy* was 92.3% (SD 3.33) and the descriptive mean *recall* was 88.0% (SD 6.93). The descriptive mean *precision* was 89.6% (SD 6.72) and the descriptive mean *Dice coefficient* was 88.4% (SD 4.88). IoU was reported in a single study (84.6%). See Table 2 and Figure 2. (For a collection of results, see Supplement S3).

### 3.3. Burn Depth

**Figure 3:**
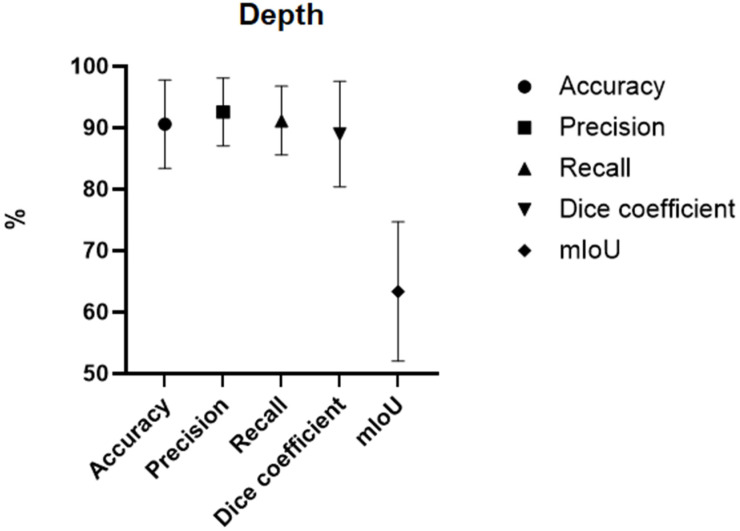
Error-bar chart showing descriptive mean values and standard deviations (SD) of reported metrics across included studies for burn TBSA/area tasks. Bars represent between-study variability in reported values and do not represent uncertainty, confidence intervals or model-level precision.

A total of 15,781 images across 17 studies were collected and processed to help diagnose the burn depth [11,19,20,21,22,23,24,25,26,27,28,29,30,31,32,33,34]. Of these images, 184 were LDPI images and 9323 images were obtained through expansion of the existing dataset. LDPI captures physiologic perfusion-related signals and is not directly comparable with RGB photography. Reported performance values across LDPI and RGB studies should therefore not be interpreted as comparative. Similarly to burn area tasks, studies reported multiple metrics. Reported descriptive means and ranges in this review span multiple imaging modalities and study designs, and they are presented for overview purposes only. *Accuracy* was the most reported metric. The mean *accuracy* attained was 90.24 with a standard deviation of ±7.61. Table 3 and Figure 3. (For a collection of results, see Supplement S3)

### 3.4. Treatment

The studies that focused on outcomes beyond TBSA and depth were grouped as treatment-related prediction tasks, including surgery vs. non-surgery, graft vs. non-graft and healing-time category prediction. In this category, a new column is introduced because of the different outcomes that can be incorporated in the broader meaning of treatment. Outcomes included in the treatment category are surgical vs. non-surgical, graft vs. non-graft and healing time with focus on depth and colour tracking (Table 4).

### 3.5. Large Language Model Evaluation

#### 3.5.1. ChatGPT

**Figure 4:**
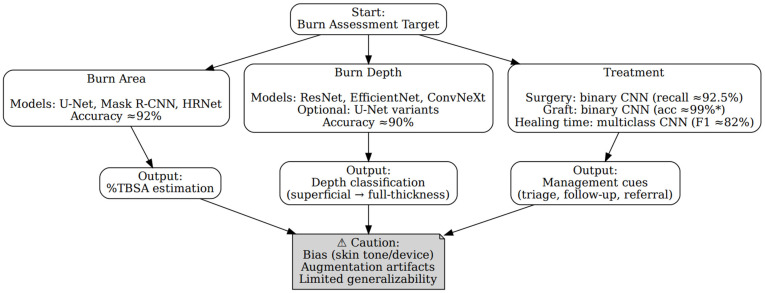
LLM-derived decision tree for AI in burn assessment. The figure summarizes reported application domains of convolutional neural networks in burn assessment, including burn area estimation, burn depth classification, and treatment-related classification tasks. Performance metrics shown reflect results reported in individual studies and are not pooled estimates. The asterisk (*) indicates values de-rived from single-study evaluations with restricted conditions, including binary outcomes, limited test sets, extensive data augmentation, and absence of external validation. The figure provides descriptive orientation of the literature and does not represent clinical decision support. Abbreviations: CNN = convolutional neural network, %TBSA = percent total body surface area, F1 = harmonic mean of precision and recall. * = this is a single-study test-set accuracy reported in our dataset, obtained from a heavily augmented/expanded training set and not pooled across studies; interpret cautiously and do not generalize.

Using the extracted evidence from the scoping review, ChatGPT produced a concise decision tree that begins with the user’s burn assessment target and branches to the model that was most used and their typical outputs (Figure 4). For burn area, segmentation backbones such as U-Net, Mask R-CNN and HRNet were linked to automated %TBSA estimation, with a descriptive mean accuracy ≈ 92% across the included studies. For burn depth, classifiers such as ResNet, EfficientNet and ConvNeXt (with optional U-Net variants for pixel-level maps) were linked to depth classification (superficial to full-thickness burn) with descriptive mean accuracy ≈ 90%. For treatment-oriented tasks, binary CNNs were linked to surgery vs. no surgery (recall ≈ 92.5% in one study) and graft vs. no graft (accuracy ≈ 99% in one study, largely augmentation-dependent), while multiclass CNNs were linked to healing-time categories (F1 ≈ 82%). The tree ends in a caution box summarizing cross-cutting limitations (dataset bias by skin tone/device, augmentation artefacts, limited external validation). These values and models reproduce our tabulated findings and do not constitute clinical recommendations.

Starting from the user’s burn assessment target, tree branches lead to three domains that show which model is most commonly used in our review and the typical outputs they produce. Burn area: representative segmentation backbones (U-Net, Mask R-CNN, HRNet) with outputs %TBSA estimation, descriptive mean accuracy ≈ 92% in our dataset. Burn depth: representative classifiers (ResNet, EfficientNet, ConvNeXt, optional U-Net variants for pixel-level depth maps), with output depth classification (superficial to full thickness) and descriptive mean accuracy ≈ 90%. Treatment: task-specific models with outputs, surgery vs. no surgery (binary CNN, recall ≈ 92.5%), graft vs. no graft (binary CNN, accuracy ≈ 99%*) and healing-time category (multiclass CNN, F1 ≈ 82%). The bottom caution box summarizes cross-cutting limitations observed across studies: dataset bias (e.g., skin tone/device), augmentation artefacts and limited external validation/generalizability. All models and performance values shown are taken from the included studies and our descriptive summaries; no new training or estimates were produced by ChatGPT, which only organized our extracted results.

#### 3.5.2. Evaluation of the LLM Decision Tree

Two consultant burn surgeons (J.Z., F.H.) reviewed the decision tree using the three-item form in Supplement S2. Both reviewers answered yes to all items. This yields perfect agreement on clarity, relevance and perceived usefulness. It suggests the figure communicates the intended structure and outputs without ambiguity. It also indicates that outputs match clinical expectations for area, depth and treatment. Both surgeons also evaluated the tree—it could potentially guide model choice at the level of an orientation aid.

## 4. Discussion

This scoping review mapped how convolutional neural networks (CNNs) are used for burn assessment. We focused on TBSA, burn depth and treatment-related tasks. We summarized reported performance, common model types and limitations for clinical translation. Across the included studies, reported performance for area and depth tasks was often high and treatment-related prediction tasks showed early promise. High reported performance does not necessarily imply clinical robustness or real-world accuracy, since many studies used small datasets, heavy augmentation or synthetic image generation and internal-only validation without external testing. We refined these findings by entering them into an LLM-derived orientation tree to aid readers in matching targets to typical model approaches. However, since the studies used different datasets, labels, and validation methods, the results may not generalize well. This variability likely also explains why overlap metrics like mIoU look weaker than others. Several factors likely inflate reported performance and limit transfer to routine clinical use. Many studies relied on small single-centre datasets, heavy augmentation or synthetic image generation and internal test-sets drawn from the same distribution as the training data. These design choices increase the risk of overfitting and optimistic test performance. External validation across centres, devices and skin tones was uncommon. This limitation is especially important for treatment-oriented tasks where errors affect operative decisions. Single-study results with heavily expanded datasets, such as graft versus non-graft prediction, should therefore be interpreted cautiously.

Cirillo et al. 2019 [24] compared multiple CNN architectures for burn depth assessment and reported accuracy ranging from 77.79% to 81.66%, with ResNet-101 performing highest in their study. A year later, Khan et al. 2020 [31] managed to achieve an accuracy of 79.4% when assessing burn depth. He also stated that these were the best results compared to previous studies and results. More recent studies report higher values, but heterogeneity in datasets, labels and validation limits comparability. Most of the authors state that CNNs are ready to be implemented in healthcare and that, if the performance increase continues to follow the path of the last recent years into the coming years, there is potential for CNNs to act as a valuable tool in burn healthcare. Boissin et al. 2023 [17] compared performance across lighter and darker skin groups using images from Sweden and South Africa. They reported higher recall in darker skin than in lighter skin. This finding highlights two issues. First, model performance can differ across skin tone groups due to contrast, lighting, camera properties and label noise. Second, many datasets remain limited in skin tone diversity, which raises fairness and safety concerns if models are deployed without representative training and external validation. Future studies should report skin tone distribution, device characteristics and subgroup performance to support safer translation.

As described before, the mean *Intersection over Union*, mIoU, is considerably lower than every other tested metric. Various reasons could cause this, with the first and most prominent being the limited data on the metric. In the data extraction, only one study, Zhang et al. [26], was found to measure the mIoU. This makes the interpretation of the results harder and increases the risk for statistical distortion as mean values and confidence intervals cannot be calculated. The study in question also raised concerns about their limited dataset due to an absence of publicly available datasets and sole reliance on a single internal dataset. Another explanation for this could be the metric itself and its characteristics. Müller et al. [38] explain that the metric tends to be less forgiving. This indicates that a few low values are enough to decrease the mean by a higher amount than other metrics used for the same analysis, like the *Dice coefficient*.

Regarding the treatment aspect of burns, there are fewer studies and each of these addresses a different aspect of burn management. As for the ability to forecast whether surgery is needed or not, Boissin et al. [17] shows a high recall of 92.5%. Yadav et al. [33] focused on graft vs. non-graft reaching a 99.67% accuracy. Both studies’ outcomes may be explained by the usage of a simpler binary classification that allows for less error. Wang et al. [35] assessed the healing time by examining if the burn was shallow/superficial (0–10 days to healing), moderate (11–20 days), deep (more than 21 days) or needed skin graft, showing a recall of 82.34%. Another study addressing healing time, Ethier et al. [22], did so by assessing the colours of the burn where red was granulation/inflammation, yellow was slough, black was necrotic tissue and white was scabbing or epithelialization. Binary models work well for triage as a quick, accurate first-pass filter; multiclass models can support more detailed assessments in complex clinical situations. CNNs seem to have the potential to support the future of burn care; this is shown by their achieved results across multiple studies. Despite the positive results, limitations exist and improvements can be made. Large datasets can create new relevant ethical considerations, such as ensuring data protection with increasing digitalization. Still, studies need to increase and diversify the training of the models, so they are weighted and properly generalized in order not to suffer when applied to unseen data. This is especially important as many studies include the integration of mobile platforms. Analyzing and drawing conclusions is currently difficult due to methodological differences between the studies. To combat this hurdle, future studies should strive for more standardized methods and outcome parameters to make comparisons and larger-scale meta-analyses possible.

### Limitations

This scoping review includes studies with substantial heterogeneity in datasets, skin tone distribution, imaging modality, augmentation strategy, model type and reported metrics. Many studies used different outcome definitions and validation approaches. For this reason, the descriptive means and SDs reported here do not represent pooled performance and do not support direct cross-study comparison. The values should be read as an overview of reported results rather than as benchmarks for model selection or clinical deployment. Some included studies used LDPI-based imaging or other physiologic modalities, while others used RGB photography from smartphones or clinical cameras. These modalities capture different signals and are not directly comparable. Mixing modality-specific results can bias the interpretation of performance values and clinical transfer. We therefore emphasize modality as a key limitation when interpreting reported metrics and generalizability.

This review has several limitations. Many of the included studies relied on small datasets, often expanded through augmentation or GAN-generated images, which may introduce unrealistic features and reduce external validity. The reference standards used for labelling, such as LDPI and clinical assessment, are themselves imperfect, which could reduce the reliability of the models. Conflicts of interest were inconsistently reported, reducing transparency. Finally, the restriction to only three databases for the search may have limited coverage. The decision tree generated by the LLM has several limitations. It inherits all biases and errors present in the underlying studies (e.g., small, single-centre datasets, variable labels, heterogeneous validation) and adds additional constraints. One of them is comparability; the performance numbers come from different tasks’ datasets, splits and metrics and are not directly comparable. Abstraction is another constraint, as the figure simplifies diverse architectures and training schemes into a decision tree. LLMs can misread and/or over-generalise summaries. The tree has also not been validated prospectively and must not guide patient care. The LLM only used values extracted in this review and obtained an independent review by two consultant burn surgeons; nonetheless, any recommendation by this tree should be interpreted cautiously and verified in prospective studies. In accordance with PRISMA-ScR guidance, a formal risk-of-bias assessment was not performed.

## 5. Conclusions

Current studies have proven the inclusion of AI and CNNs to be promising, achieving high results in diagnostic metrics for burn assessment. With more standardized methods and outcome parameters in the future to minimize their limitations, CNNs can function as a solution to many of the limitations of current methods of assessment. The clinical value of CNNs in burn care remains to be established through standardized datasets and external validation. The LLM-derived decision tree could potentially be used as an orientation aid, not as a clinical tool in burn assessment.

## Figures and Tables

**Table 1:** Summary of data extraction. Table shows the distribution of the included studies. Studies are divided into the used design and reported outcome.

Studies	24
Design	
Comparative	12
Experimental	12
Reported outcome	
Burn area (%TBSA)	10
Burn depth assessment	17
Treatment	4

**Table 2:** Data extraction from the studies that included burn area. Information about the studies is described in the columns in a descriptive and numerical format. The column AI model includes information about the name of each convolutional network used, including a short description. If other than patient images were used, this is stated in the dataset and training set columns. #= number of.

Author/Title/Year	Country	Study Design	AI Model	Dataset (# of Images)	Training Set (# of Images)	Summary Statistics
Dai et al. 2021 [14] Burn Images Segmentation Based on Burn-GAN	China	Comparative study	Burn-GAN architecture that synthesizes realistic images	1150	1000 + 960 generated	Accuracy = 96.88% Precision = 90.75% DC = 84.5% to 89.3%
Chang et al. 2021 [15] Deep Learning-Assisted Burn Wound Diagnosis: Diagnostic Model Development Study.	Taiwan	Comparative study	U-Net or Mask R-CNN with ResNet50 or ResNet101	2591	2073 (8:1:1, 80% train, 10%validation, 10% test.)	Accuracy = 91.30% Precision = 96.13% Recall = 93.90%
Chauhan et al. 2021 [16] Convolution neural network for effective burn region segmentation of colour images.	India	Experimental study	ResNet101	434	316	Accuracy = 93.36% Precision = 81.95% Recall = 83.39 Specificity = 95.70% DC = 81.42%
Boissin et al. 2023 [17] Development and evaluation of deep learning algorithms for assessment of acute burns and the need for surgery.	Sweden, South Africa	Experimental study	Aiforia Create self-service deep learning tool	1105 + 536 background	773	Accuracy = 86.9% Precision = 83.4% DC = 82.9% Recall Darker Skin = 89.3% Recall Lighter skin = 78.6%
Liu et al. 2021 [11] A Framework for Automatic Burn Image Segmentation and Burn Depth Diagnosis Using Deep Learning.	China	Experimental study	ResNet-50 with ResNEt-101 modified to HRNetV2	516 + 1200 expanded	960	IOU = 84.67% DC = 91.70%
Xu et al. 2024 [18] On-site burn severity assessment using smartphone-captured color burn wound images.	China	Comparative study	ResNet and ResNeXt modified to ConvNeXt	917	Evaluated with 6-fold cross-validation	DC = 85.36% R^2^ for %TBSA = 91.36%
Abdolahnejad et al. 2025 [19] Novel CNN-Based Approach for Burn Severity Assessment and Fine-Grained Boundary Segmentation in Burn Images	Canada	Experimental study	EfficientNet B7	1385 + 184 LDPI images	1385	Accuracy = 91.39%
Pabitha et al. 2021 [20] Densemask RCNN: A Hybrid Model for Skin Burn Image Classification and Severity Grading	India	Comparative study	ResNet-101 and DenseMask RCNN	1800	1200	DC = 87.10%
Pabitha et al. 2024 [37] Dense Mesh RCNN: assessment of human skin burn and burn depth severity	India	Comparative study	ResNet-101 and Dense Mesh RCNN	1150	1000	Accuracy = 94.10% Precision 95.90% Recall = 94.80% DC = 95.30%

**Table 3:** Data extraction from the studies that included burn depth. Information about the studies is described in the columns in a descriptive and numerical format. The column AI model includes information about the name of each convolutional network used, including a short description. If other than patient images were used, this is stated in the dataset and training set columns. #=number.

Author/Title/Year	Country	Study Design	AI Model	Dataset (# Images)	Training Set (# Images)	Summary Statistics
Ethier et al. 2024 [22] Using Computer Vision and Artificial Intelligence to Track the Healing of Severe Burns.	Canada	Experimental study	EfficientNet B7	1559	1285	Recall = 82% Precision = 83% DC = 82%
Lee et al. 2025 [23] Comparing Artificial Intelligence Guided Image Assessment to Current Methods of Burn Assessment.	England	Comparative study	EfficientNet B7 modified integration of Boundary attention mapping (BAM)	1868	1684	Area under the curve (AUC) = 85%
Cirillo et al. 2019 [24] Time-Independent Prediction of Burn Depth Using Deep Convolutional Neural Networks.	England	Comparative study	ResNet50 ResNet101	23 expanded to 676	10-fold cross-validation	Accuracy ResNet50 = 77.79% ResNet101 = 81.66%
Li et al. 2023 [25] GL-FusionNet: Fusing global and local features to classify deep and superficial partial thickness burn.	China	Comparative study	U-Net With fusion of ResNet50 and ResNet101	500 expanded to 3264	5-fold cross-validation	Accuracy = 93.52% Recall = 93.67% Precision = 93.51% DC = 93.51%
Liu et al. 2021 [11] A Framework for Automatic Burn Image Segmentation and Burn Depth Diagnosis Using Deep Learning.	China	Experimental study	ResNet-50 with ResNet-101 modified to HRNetV2	516 expanded to 1200.	960	IoU = 51.44% Accuracy = 66.84% DC = 68.82%
Zhang et al. 2025 [26] Semi-Supervised Burn Depth Segmentation Network with Contrast Learning and Uncertainty Correction.	China	Comparative study	U-Net modified to semi-supervised model SBCU-net	1142	914	50% labelled data: Accuracy = 94.32% DC = 84.51% mIoU = 74.04% 10% labelled data Accuracy = 92.10% DC = 76.95% mIoU = 64.58%
Cirillo et al. 2021 [27] Improving burn depth assessment for paediatric scalds by AI based on semantic segmentation of polarized light photography images.	Sweden, Saudi Arabia	Experimental study	Modified U-Net	100	16	Accuracy = 91.89% DC = 91.88%
Pabitha et al. 2022 [28] FASTER-RCNN for Skin Burn Analysis and Tissue Regeneration	India	Comparative study	R-CNN modified to Faster RCNN	1300	1000	Recall = 89.60% Precision = 98.46% DC = 95.20%
Abdolahnejad et al. 2025 [19] Novel CNN-Based Approach for Burn Severity Assessment and Fine-Grained Boundary Segmentation in Burn Images	Canada	Experimental study	ImageNet modified to EfficientNet B7 EfficientNet B7	1385 + 184 LDPI images	1385	Accuracy 80%
Suha et al. 2022 [29] A deep convolutional neural network-based approach for detecting burn severity from skin burn images	Bangladesh	Experimental study	VGG16	1530	1071 images	Accuracy = 95.80% Recall = 95.00% Precision = 96.00% DC = 95.00%
Pabitha et al. 2021 [20] Densemask RCNN: A Hybrid Model for Skin Burn Image Classification and Severity Grading	India	Comparative study	DenseMask RCNN	1800	1200	Accuracy = 86.63% Precision = 85.00% Recall = 89.00% DC = 86.90%
Abubakar et al. 2020 [30] Assessment of Human Skin Burns: A Deep Transfer Learning Approach	UK, Nigeria	Experimental study	ResNet50	1360 UK cohort + 540 Nigerian cohort	1520	Accuracy = 97.10% using the Nigerian dataset Accuracy = 99.30% using the Caucasian dataset
Pabitha et al. 2024 [21] Dense Mesh RCNN: assessment of human skin burn and burn depth severity	India	Comparative study	Dense Mesh RCNN	1150	1000	Accuracy = 94.10% Precision 95.90% Recall = 94.80% DC = 95.30%
Khan et al. 2020 [31] Computer-aided diagnosis for burnt skin images using deep convolutional neural network	Pakistan	Experimental study	CNN modified with more layers to DCNN	450	293	Accuracy = 79.40%
Abubakar et al. 2020 [32] Burns Depth Assessment Using Deep Learning Features	UK, Nigeria	Comparative study	ResFeat50, VggFeat16	2080	520 LDPI images	ResFeat50 Accuracy = 95.43% Precision = 95.50% Recall = 95.50% DC = 95.50% VGGFeat16 Accuracy = 85.67% Precision = 86.25% Recall = 85.75% DC = 85.75%
Yadav et al. 2022 [33] Human burn depth and grafting prognosis using ResNeXt topology based deep learning network	India	Experimental study	ResNeXt modified to BNeXt	94 expanded to 6000	4800	Accuracy = 97.17% Recall = 97.25% Precision = 97.20% DC = 97.22%
Yadav et al. 2023 [34] Spatial attention-based residual network for human burn identification and classification	India	Experimental study	ResNeXt modified to BuRnGANeXt50	94 expanded to 6000	4800	Accuracy = 98.14% Recall = 97.22% Precision = 97.22% DC = 97.22%

DCNN: deep convolutional neural network.

**Table 4:** Data extraction from the studies that include treatment aspects with the use of CNNs. Information about the studies is described in the columns in a descriptive and numerical format. The column AI model includes information about the name of each convolutional network used, including a short description. If other than patient images were used, this is stated in the dataset and training set columns. The outcome column describes specifics about the outcome of each study. # = number of.

Author/Title/Year	Country	Study Design	AI Model	Data Set (# Images)	Training Set (# Images)	Outcome	Summary Statistics
Wang et al. 2020 [35] Real-time burn depth assessment using artificial networks: a large-scale, multicentre study.	China	Experimental study	ResNet50	484 expanded to 5637 images	3945	Healing time Shallow (0–10 days), moderate (11–20 days), deep more than 21 days or wound healing by surgical skin graft	Recall = 82.34% Precision = 82.67% F1 score = 82%
Ethier et al. 2024 [22] Using Computer Vision and Artificial Intelligence to Track the Healing of Severe Burns.	Canada	Experimental study	Skin abnormality tracking algorithm that uses BAM	1559	1285	Healing Time By tracking 4 colours of the wound	F1 Score 82%
Boissin et al. 2023 [17] Development and evaluation of deep learning algorithms for assessment of acute burns and the need for surgery.	Sweden, South Africa	Experimental study	Aiforia Create self-service deep learning tool	1105 + 536 background images	773	Surgical vs. non-surgical	Recall = 92.5% Specificity = 53.6% AUC = 88.5%
Yadav 2022 [33] Human burn depth and grafting prognosis using ResNeXt topology based deep learning network	India	Experimental study	Modified version of ResNeXt to BNeXt	94 expanded to 6000	4800	Graft vs. non-graft	Accuracy = 99.67%

## Data Availability

The original contributions presented in this study are included in the article/Supplementary Material. Further inquiries can be directed to the corresponding author(s).

## Supplementary Materials

The following supporting information can be downloaded at: https://www.mdpi.com/article/10.3390/ebj7010004/s1. Supplement S1: A display of our search strategies; Supplement S2: Template for the Evaluation of AI Decision Tree (Burn Assessment) by the two consultants; Supplement S3: A compilation of our collected results. This was used in the calculation of mean values and standard deviations. Made in Microsoft Excel; Supplement S4: The prompt provided to ChatGPT to create the generated decision tree.
