# Supplementary material for: Use of Artificial Intelligence in Burn Assessment: A Scoping Review with a Large Language Model-Generated Decision Tree
Source: Eur Burn J. 2026 Jan 4;7(1):4. doi: 10.3390/ebj7010004 (PMC12821601; doi:10.3390/ebj7010004)
Supplement: Supplementary file 1 [file ebj-07-00004-s001.zip › ebj-4029380-supplementary.pdf]

Supplementary material

Supplement S1. A display of our search strategies

PubMed

#	Search term	Amount	Category
1	**Burns [MeSH]**	64,581	Burn-related
2	"Burn injur*"[Title/Abstract]	12,216	Burn-related
3	"Burn wound"[Title/Abstract]	4,367	Burn-related5
4	"Burn depth*"[Title/Abstract]	591	Burn-related
5	"Thermal injur*"[Title/Abstract]	6,192	Burn-related
6	"superficial burn"[Title/Abstract]	102	Burn-related
7	"skin burn*"[Title/Abstract]	1,681	Burn-related
8	Combined search of burns (OR)	71,645	Burn-related
9	**"Artificial intelligence" [MeSH]**	229,665	AI-related
10	"image segmentation"[Title/Abstract]	9,704	AI-related
11	"image analysis"[Title/Abstract]	48,368	AI-related
12	"computer vision"[Title/Abstract]	11,193	AI-related
13	"computer reasoning"[Title/Abstract]	8	AI-related
14	"computer vision system"[Title/Abstract]	273	AI-related

15	"computational intelligence"[Title/Abstract]	597	AI-related
16	"machine intelligence"[Title/Abstract]	389	AI-related
17	“machine learning”[Title/Abstract]]	144,022	AI-related
18	“neural network”[Title/Abstract]	89,841	AI-related
19	Combined AI search (OR)	444,800	AI-related
20	**Combined search (Burns AND AI)**	268	Combination

Web of Science - 376 Documents

TS=("Artificial Intelligence" OR "Computer Vision" OR "image analysis" OR "image segmentation" OR "deep learning" OR "machine learning" OR "neural network*")

AND

TS=("Burns" OR "burn injury" OR "burn wound" OR "burn depth" OR "thermal injur*" OR "skin burn*" OR "superficial burn*")

Cochrane

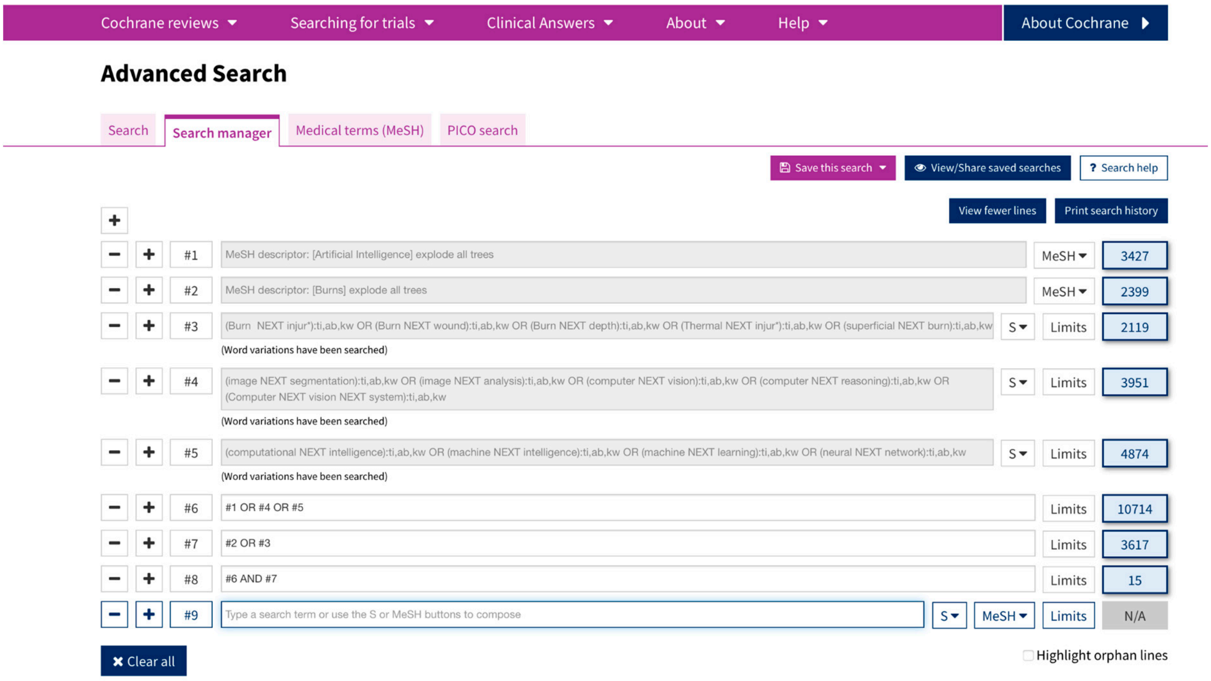

Supplement S2. Template for the Evaluation of AI Decision Tree (Burn Assessment) by the two consultants.

Evaluation of AI Decision Tree (Burn Assessment)

Please review the decision tree. For each question, mark one option.

1. Clarity – Is the decision tree easy to understand?

☐ Yes ☐ No

2. Relevance of outputs – Are the recommended outputs (e.g., %TBSA, depth classification, treatment cues) reasonable in relation to the suggested AI models?

☐ Yes ☐ No

3. Usefulness – Could this decision tree be used to guide which AI model is recommended depending on the burn assessment target (area, depth, treatment)?

☐ Yes ☐ No

Optional comments (1–2 sentences):

Supplement S3. A compilation of our collected results. This was used in the calculation of mean values and standard deviations. Made in Microsoft Excel

**Burn area**	**Accuracy**	**Precision**	**Recall**	**Dice coefficient**	**Specificity**	**IoU**
	94,1	90,75	93,9	81,42	81,95	84,67
	91,39	96,13	83,39	91,7		
	86,9	81,95	89,3	87,1		
	93,36	83,4	78,6	95,3		
	91,3	95,9	94,8	89,3		
	96,8			85,36		
Average	92,3083333	89,626	87,998	88,36333333	81,95	88,67
SD	3,33470488	6,71960788	6,93455262	4,877580001		

**Burn depth**	Accuracy	Precision	Recall	Dice coefficient	mIoU
	92,1	83	82	82	51,44
	94,32	93,51	93,67	93,51	74,04
	77,79			68,82	64,58
	81,66	98,46	89,6	84,51	
	93,52	96	95	76,95	
	91,89	85	89	91,88	

	80	95,9	94,8	95,2	
	95,8	95,5	95,5	95	
	86,63	86,25	85,75	86,9	
	97,1	97,2	97,25	95,3	
	99,3	97,22	97,22	95,5	
	94,1			85,75	
	79,4			97,22	
	95,43			97,22	
	85,67				
	97,17				
	98,14				
Average	90,5894118	92,804	91,979	88,98285714	63,3533333 3
SD	7,19695202	5,75768511	5,16943893	8,578661747	11,34982526

Supplement S4. The prompt provided to ChatGPT to create the generated decision tree.

You are provided with a structured summary of published studies evaluating convolutional neural networks for burn assessment. The information includes task domain (burn TBSA/are, burn depth, treatment-related tasks, imaging modality (RGB photography, LDPI), model type as reported by the authors and reported performance metrics. Your task is to organize this information into a one-page, high-level orientation decision tree that summarizes how CNNs have been applied across burn assessment tasks. Use only information provided. Do not generate new performance metrics, estimates or claims. Do not extrapolate beyond the reported data. Do not provide clinical recommendations. The output should serve as a literature-based visual summary to help readers understand how existing studies structures across tasks are and modalities. The output is not intended as a clinical decision-support tool.
